# Open Science in Africa: What policymakers should consider

**DOI:** 10.3389/frma.2022.950139

**Published:** 2022-11-03

**Authors:** Elisha R. T. Chiware, Lara Skelly

**Affiliations:** ^1^Cape Peninsula University of Technology Library, Cape Peninsula University of Technology, Cape Town, South Africa; ^2^Loughborough University Library, Loughborough University, Loughborough, United Kingdom; ^3^Stellenbosch Business School, Stellenbosch University, Stellenbosch, South Africa

**Keywords:** Africa, Open Science policy, Open Science, policy development, Open Access, Open Data

## Abstract

As Open Science (OS) is being promoted as the best avenue to share and drive scientific discoveries at much lower costs and in transparent and credible ways, it is imperative that African governments and institutions take advantage of the momentum and build research infrastructures that are responsive to this movement. This paper aims to provide useful insight into the importance and implementation of OS policy frameworks. The paper uses a systematic review approach to review existing literature and analyse global OS policy development documents. The approach includes a review of existing OS policy frameworks that can guide similar work by African governments and institutions. This critical review also makes recommendations on key issues that Africa should consider in the process of OS policy development. These approaches can be widely used as further foundations for future developments in OS practices on the continent.

## Introduction

The importance of promoting Open Science (OS) as the vision for the future of conducting science is shared by many, and it is gaining momentum across institutions, governments, and regions at a global level. In different areas–especially in Europe, the US, the UK, and Canada–governments have moved to create the necessary national OS policy frameworks to guide how institutions should respond to the call. The roadmaps toward the OS vision are being shaped by guiding principles such as Open Access; the adoption of Open Data and FAIR (findable, accessible, interoperable and reusable) data principles and citizenship science; the recognition, support and training of researchers; the participation of communities; the development of infrastructures, policies and regulations; and the need for broader stakeholder engagement, coordination and high-level government support (Boulton et al., 2020; Burgelman, 2021; Clark, 2021; Manco, 2021).

In 2021, the United Nations Educational, Scientific and Cultural Organization (UNESCO) provided a set of recommendations as “an international framework for OS policy and practice that recognizes disciplinary and regional differences in OS perspectives, takes into account academic freedom, gender-transformative approaches and the specific challenges of scientists and other Open Science actors in different countries and in particular in developing countries, and contributes to reducing the digital, technological and knowledge divides existing between and within countries” (UNESCO, 2021). These recommendations are also seen as a support mechanism for a global response to fulfilling the Sustainable Development Goals (SDGs), especially among poorer countries in Africa, Latin America, and Asia. The principles of OS, which include the FAIR and open sharing of scientific research outputs, including data, are seen as an anchor to solving health, developmental, educational and social problems in a more coordinated way (Mwelwa et al., 2020; Abebe et al., 2021; UNESCO, 2021).

Concerns have been raised about the violation of some of the OS principles and its potential impact on the quality of research output during the COVID-19 pandemic–hence a call for “a wider adoption of OS practices in the hope that this work will encourage a broader endorsement of OS principles and serve as a reminder that science should always be a rigorous process, reliable and transparent, especially in the context of a pandemic where research findings are being translated into practice even more rapidly” (Besançon et al., 2021, p. 1). The Organization for Economic Co-operation and Development (OECD) also argued that “in global emergencies like the coronavirus (COVID-19) pandemic, OS policies can remove obstacles to the free flow of research data and ideas, and thus accelerate the pace of research critical to combating the disease” (OECD, 2020).

Drawing on the UNESCO recommendations and emerging research on the critical role of OS principles, there are many opportunities for African institutions and governments to shape their own roadmaps on OS through the development of research infrastructures and supporting, responsive and coordinated policy frameworks at national and institutional levels (Mwelwa et al., 2020). The OECD emphasizes that “to strengthen the contribution of OS to the COVID-19 response, for example, policymakers need to ensure adequate data governance models, interoperable standards, sustainable data-sharing agreements involving the public sector, private sector and civil society, provide incentives for researchers, build sustainable infrastructures, develop human and institutional capabilities and mechanisms for access to data across borders” (OECD, 2020). These principles apply to all other global developmental challenges hampered by climate change, energy provision, and lack of equity in education and other social services.

Reporting on the state of Open Science in research and innovation for development in sub- Saharan Africa, Boulton et al. (2020, p. 7) observed that “the basis of the OS revolution and its impacts” will leave Africa with no alternatives but to respond to its challenges. The value of an OS environment in Africa should be based on two fundamental premises: first, that data sharing and access to scientific data is affordable and easy, and second, that OS engages with society, business, policymakers, governments, communities and citizens as knowledge partners in ways that increase both effectiveness and socio-political legitimacy (Mwelwa et al., 2020).

Emerging continental, regional and national bodies and African programmes are working toward OS's development goals (Boulton et al., 2020; Chiware, 2020; Mwelwa et al., 2020; Abebe et al., 2021). One of the most ambitious projects is the African Open Science Platform, whose mission is to put African scientists at the cutting edge of contemporary, data-intensive science as a fundamental resource for modern society. Its building blocks are federated hardware, communications and software infrastructure, including policies and enabling practices to support OS in the digital era; and a network of excellence in OS that supports scientists and other societal actors in accumulating and using modern data resources to maximize scientific, social and economic benefit (Smith and Veldsman, 2018).

Another important continental initiative toward the development of OS is LIBSENSE (2022), launched in 2016 to bring together the research and education networks (RENs) and academic library communities to strengthen OS in Africa. LIBSENSE provides an avenue through which different stakeholder communities can collaborate to define priority activities, share knowledge, and develop relevant services. LIBSENSE is led by the West and Central African Research and Education Network (WACREN) in collaboration with sister regional African RENs (ASREN and UbuntuNet Alliance). Other participating partners include several national RENs, libraries, library associations, universities and research communities in Africa, in conjunction with COAR, EIFL, University of Sheffield, National Institute of Informatics (Japan), GEANT, and OpenAIRE. Outcomes of the LIBSENSE initiative include metadata guidelines for repositories, plans for a regional repository hosting service, and national and institutional policy templates (COAR).

Leading international library and information services organizations are pivotal in enabling African institutions to engage in OS policy development. For example, Electronic Information for Libraries (EIFL, 2022), a not-for-profit organization, works with libraries to allow access to knowledge in developing and transition economy countries in Africa, Asia Pacific, Europe and Latin America. In Africa, EIFL has partnered with library consortia in countries like Ethiopia, Kenya, Uganda, and Zimbabwe and has launched projects to boost open access and OS policy development and to improve repositories and training. Botswana, Kenya, Madagascar, Mauritius, South Africa, and Uganda have progressed toward developing Open Data policies. Ethiopia is the first to have produced a national Open Access policy framework. Through collaborative dialogue with the European Union, South Africa has moved closer to finalizing a national OS framework.

Mwelwa and his fellow researchers (2020) have outlined some barriers, solutions and opportunities for OS in Africa. They have shown that the development of OS in Africa could be used to energize national science systems and enhance the roles they play in supporting the public and private sectors as well as the general public. However, they pointed out some of the barriers to achieving openness in scientific research work, which include the lack of synergies among “African science systems that largely operate independently of each other, creating silos of incompatible policies, practices and data sets that are not mutually consistent or inter-operable” (Mwelwa et al., 2020, p. 1). Abebe et al. (2021) also argued that “the future of open data management and data sharing and their contribution to the advancement of science and technology in Africa will continue to increase, despite the slow pace caused by the lack of funding, redundant policy frameworks, and limited infrastructures.” Abebe et al. (2021) explained that the African landscape is unique; the existing challenges and how they can be addressed will continue to be a big part of African participation in OS and open data global projects.

The purpose of this paper is to explore the significance of national and institutional policy frameworks in promoting OS and what policymakers should consider when developing these policy frameworks. The paper uses a systematic review approach to review existing literature and global OS policy development documents. The approach includes a review of international and national Open Science policy frameworks that can guide similar work by African governments and institutions.

## Literature review

The development of national OS policy frameworks can also be guided by the principles that the policies should respond to and support national and institutional goals in order to advance science and knowledge production and sharing. OS policy development in Africa can be framed within key principles that include open access, open data, citizenship science, collaboration, and stakeholder and community engagements. OS policy development should be clearly understood in terms of these fundamental principles of institutions and governments to achieve the end goals of openness, integrity, and FAIR data sharing within the African and global research systems.

As mentioned in the introduction, UNESCO has released draft recommendations serving as “an international framework for OS policy and practice that recognizes disciplinary and regional differences in OS perspectives, takes into account academic freedom, gender-transformative approaches and the specific challenges of scientists and other OS actors in different countries and in particular in developing countries, and contributes to reducing the digital, technological and knowledge divides existing between and within countries” (UNESCO, 2021).

In Europe, Burgelman (2021) wrote about politics and OS and how the European Open Science Cloud (EOSC) has become a reality. The establishment of EOSC is said to be one of the key results that emerged from the policy intentions to foster OS in Europe through the European Open Science Strategy. The other components of this strategy include the Open Science Policy Platform, Open Access Publishing, and the EU Citizen Science Platform. The work to achieve this can also be attributed to the long and complex history of collaboration within the European Union.

In Canada, the government released the Roadmap for Open Science, a set of principles and recommendations to guide the country's federal scientific research. The guidelines and recommendations apply to research by federally employed researchers and research contracted by federal departments and agencies. The Roadmap was developed as part of the commitment to OS as outlined in Canada's 2018–2020 National Action Plan on Open Government (Government of Canada, 2020).

The approaches show governments' and continental bodies' commitment to OS through coordinated policy frameworks that provide institutions with clear guidelines on end goals. Similar continental collaborative OS approaches have evolved in Australia, New Zealand, and the United Kingdom. Africa has the African Union (AU) with a history of policy documents that are not followed through due to member states' lack of financial commitments. Some of the promising projects rely on donor funding. Tieku (2019) pointed out that the African Union has only been able to address the needs of the political elite. The AU, he asserted, has been less successful in connecting its activities and programmes to ordinary Africans. Tieku (2019) also pointed out that the AU has been less successful in providing common public goods and services and has failed to give a voice to the majority of young people and to promote intra-Africa trade. Other areas in which the AU has not fared well include good governance, the financial independence of the continent, and the struggle to address the expressed material needs and quotidian concerns of ordinary Africans (Tieku, 2019).

Boulton et al. (2020, p. vi) recommended that “science systems in Africa...must adapt their working practices” to the “'Open Science' movement,” which has been made possible through the use of new digital technologies. This adaptation can be achieved through “the provision of IT infrastructure, policies, incentives, methods and standards for data sharing, policing of ethical standards, and systems and software needed by high-level analytic and AI procedures; such that no individual and few organizations or states in Africa could hope to provide them alone.” Boulton et al. (2020, p. vi) also encouraged an approach that has proven effective at “institutional, disciplinary, national, or international levels in scaling up the effort to develop well-managed, integrated digital services and open, sharing practices through OS platforms or commons that serve a broad community through the more or less seamless provision of support and processes for highly creative interactions.”

In Africa, Mwelwa et al. (2020) have identified one of the key barriers to OS in Africa and its institutions: the lack of policies, policy coherence, and alignment and harmonization to achieve one big goal of openness. OS practices are new approaches to doing scientific research. In many ways, these new approaches interfere with established norms–hence the need to guide its uptake through policies and guidelines at institutional and national levels. The African research environment's participation in the global OS movement rests on solid policy frameworks. There is a need to review the progress and make recommendations on how this can be achieved.

In Europe, progress toward the European Open Science Cloud and related advanced research infrastructures has been made through collaboration, policies, engagement with researchers and communities, and the promotion of citizenship science. According to Carillo and Papagni (2014, p. 42), “the production of scientific knowledge is widely recognized as one of the key factors of the economic growth which has occurred in western countries since the Industrial Revolution” and has helped to advance OS to its current levels in those environments. Carillo and Papagni (2014) also regarded the institution of “Open Science” as a cause of scientific and economic inequalities among countries. In developing countries, the limits in knowledge production due to lack of investment in research, lack of incentives, brain drain and slow pace of digital infrastructure development have primarily accounted for the slow pace of OS advancement (Chiware, 2020). Therefore, a general understanding of the politics, progress and barriers regarding Africa's adoption of OS can assist in addressing some of the challenges and bolstering good practices (Boulton et al., 2020). Abebe et al. (2021, p. 9) emphasized this point by noting that “the unique African landscape, and especially the existing challenges and how they can be addressed, will continue to play a big part in African participation in OS and open data global projects.” National and institutional policy frameworks, regulations or legislation on data sharing, access, and use are all necessary steps in enabling OS in African academic and research institutions.

## Methods and tools

The systematic review followed the protocol described by Dempster (2003), which is specific for social science research. It brought in the following elements from the Preferred Reporting Items of Systematic Reviews and Meta-Analysis checklist (Page et al., 2021) to add to clarity.

### Eligibility criteria

Results were not limited to any particular geographical area, date range or policy actor. The only criterion that was set was to include English language results only. These limitations were due to the language limitations of the researchers.

### Search strategy

The keywords used were “open research” OR “Open Science” appearing in the title field and “policy” OR “policies” appearing in the title or abstract fields. The exception for the latter was made in Google Scholar, which did not allow for searches in title *or* abstract fields, only title fields. Table 1 shows the data sources used and the number of results found.

**Table 1:** Data sources used (searches performed on 16 February 2022).

**Datasource**	**Results**
Web of science	12
EbscoHost collections: Academic Search Premier; Africa-Wide Information; Business Source Premier; CINAHL; EconLit; ERIC; GreenFILE; Health Source: Nursing/Academic Edition; Library, Information Science & Technology Abstracts; MasterFILE Premier; MEDLINE; Newspaper Source	155
Scopus	93
Google scholar	127

### Validity

Results discussing open research agendas, questions, directions or issues were excluded, as were any describing grassroots movements within disciplines. The included work had to address a particular policy actor(s). The vast majority of published scientific works mention policy as a tangential topic within conclusions. As policies were a key focus of this study, “policy” or “policies” had to appear at least five times in the body of the work. Where only the abstracts of the conference proceedings were available, they were excluded; full papers were included.

### Data extraction

**Figure 1:**
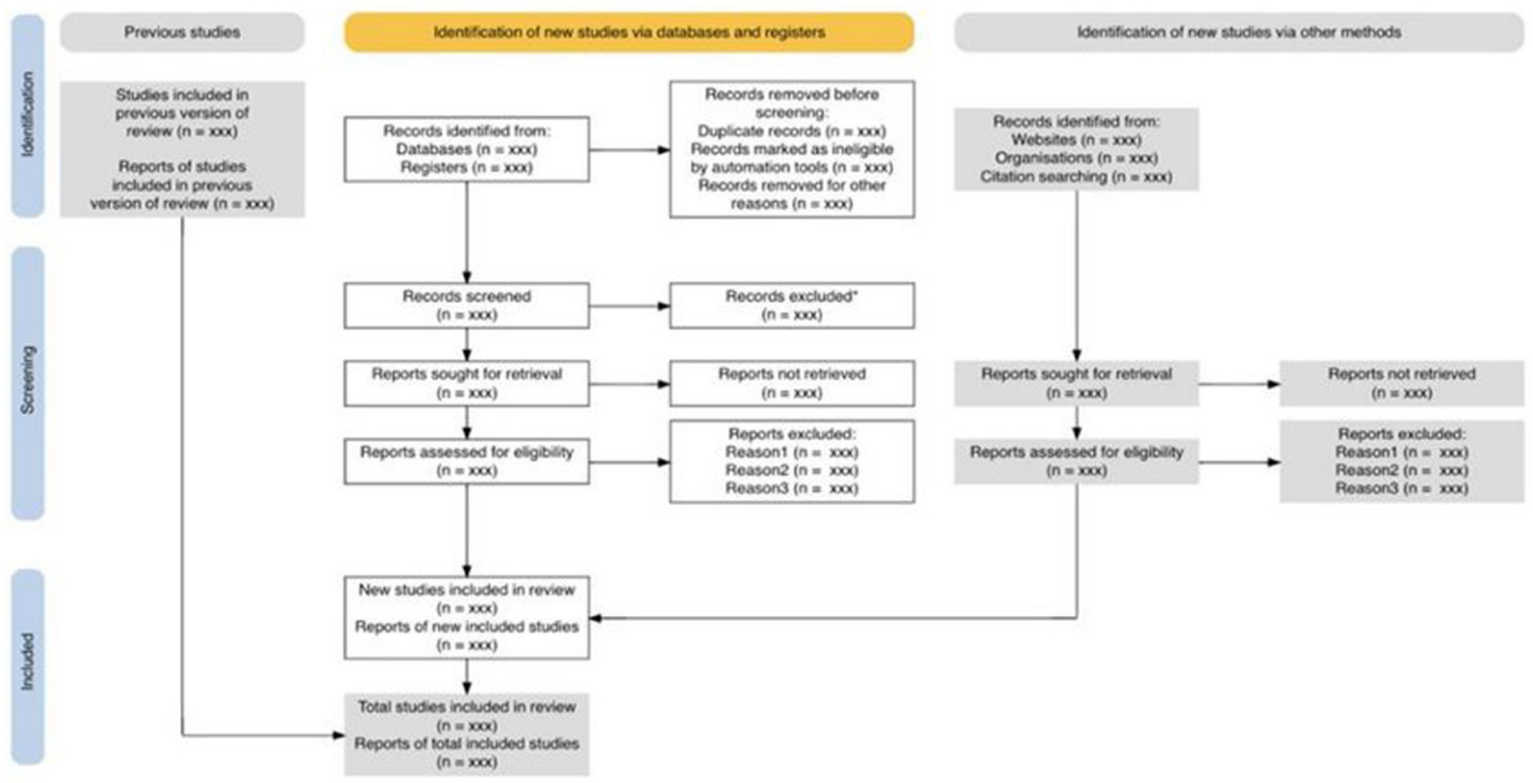
The PRISMA 2020 statement: an updated guideline for reporting systematic reviews (Adapted from Page et al., 2021).

All results were exported to Mendeley as the preferred tool to house the documents. After that, the system's deduplication function was employed. Next, the titles were screened for relevance, followed by the abstracts. Where abstracts were missing, the first paragraph was screened. After the abstract screening, full texts were searched for five mentions of “policy” or “policies” and then screened for relevance. Figure 1 shows the flow diagram.

### Synthesis methods

The included results were coded according to the scheme suggested for synthesizing the results of a systematic review of policy and practice by Snilstveit et al. (2012), who provided a three-part framework showing the importance of the policy (defining and framing the problem), examining policy examples (assessing potential policy options), and showing policy implementations (identifying policy implementation considerations for selected policy options). Parallel to the policy scheme coding, the policy actors were coded with an open coding method.

## Results

### Overview

The included papers are relatively new, with the oldest works coming from 2015. This spread reflects the relative newness of this topic in the published literature. Table 2 shows the distribution over time.

**Table 2:** Number of articles per year.

**Year**	**Number of articles**
2015	2
2016	1
2017	5
2018	4
2019	3
2020	6
2021	7
2022	1

**Figure 2:**
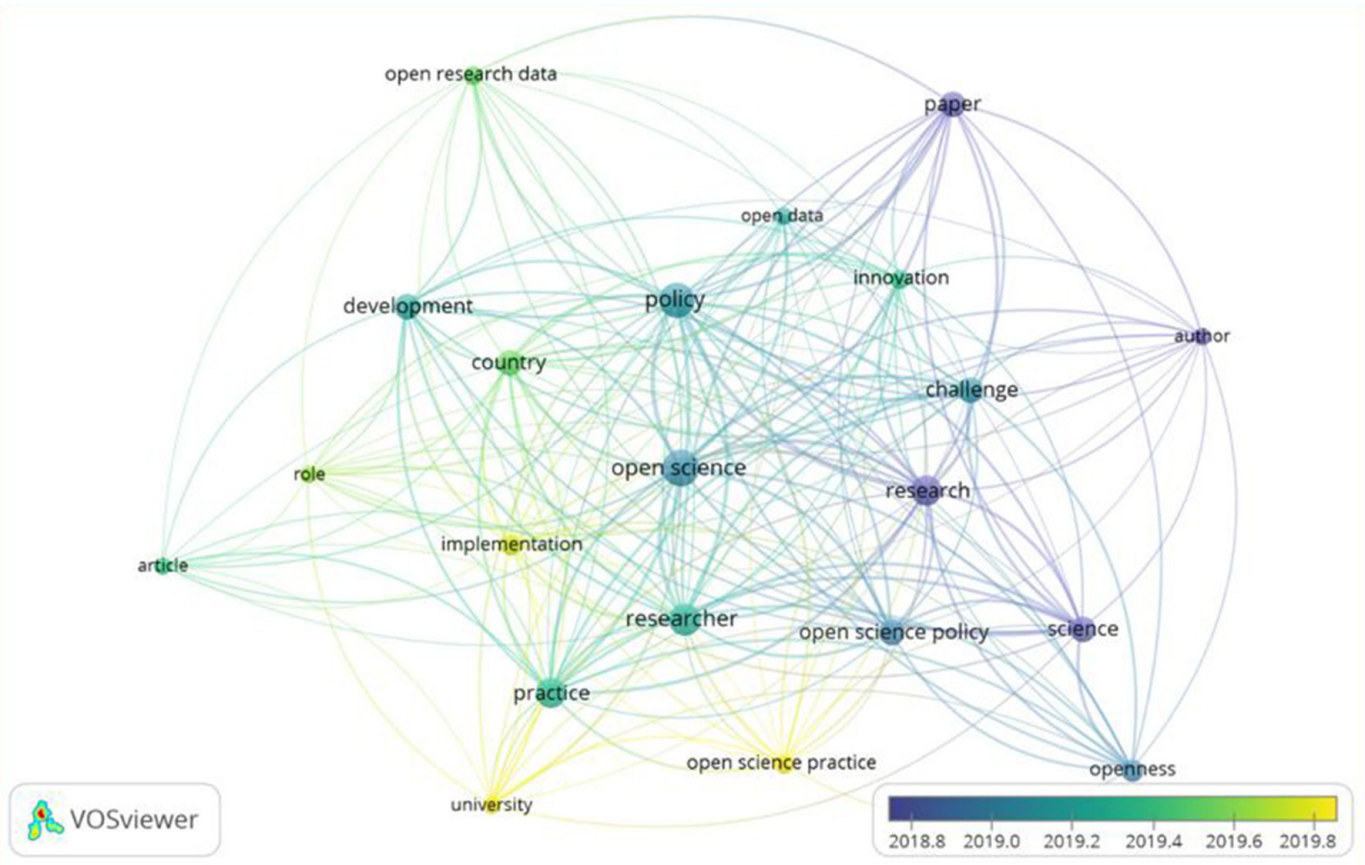
Semantic map of titles and abstracts of included articles, colors showing the average age of the keyword. VOSViewer settings: Abstract and fulltext fields; ignoring labels and statements; binary counting; minimum occurrences of terms: five; all 21 terms selected.

Despite the small time range covered by this literature review, one can see how interests in OS policies have moved among policy actors. Figure 2 is a semantic map showing that the earliest actors were authors primarily concerned with publications (papers) and that interest has shifted to an institutional level (universities) concerned with implementation. This shift reflects a certain degree of maturity in the policy landscape. That is, no longer are authors simply interested in the science; now, universities are interested in the practicalities.

The included papers come from a wide geographical spread, as shown in Table 3, with only the continents of South America and Australia not being represented.

**Table 3:** Geographical spread of papers.

**Geographic area**	**Number of articles**
International	4
Europe	3
Canada	2
China	2
Finland	2
Africa	1
Albania	1
Botswana	1
Hong Kong	1
Malaysia	1
South Africa	1
United States of America	1
No geographical region	9

Considering the currency of the topic of focus, it is not surprising that most inclusions are journal articles with a good representation of conference papers. Table 4 shows the varying publication formats of the included documents.

**Table 4:** Publication type of included papers.

**Publication type**	**Number of papers**
Conference paper	5
Journal article	16
Empirical journal article	7
Report	1

### Analysis

#### Importance

This analysis begins with framing the importance of the OS policies. The literature is abundant with details of potential or realized benefits of various OS schemes. However, in this study, only four articles could be identified that focused on setting out the importance of the open research policies.

The first of these articles is a study by Albornoz et al. (2020), showing that policies are an expression of the policy actor's values and a codification of how such values are expressed. They conceived policies as “instruments that articulate paradigms that can sustain or relocate power and legitimacy” (p. 3). Policies cannot, therefore, ever be considered neutral, OS policies included. Casting policies in this light serves as a starting point for African policymakers to enter into discussions about OS policy development. What values are the OS policies embodying? To whom are they conferring power, and from whom are they removing legitimacy? These questions could reveal possible objections or low uptake by target audiences.

Resources provide many with a solid power base, which is conferred by awarding research grants. Funders are relatively new players in the scene of OS policies. However, their importance has been growing, especially in low-resource environments where they play a significant role in funding research. The European Commission's Horizon 2020, published in 2014, mandated data management plans and open access publication (Burgelman et al., 2019). Funding was provided to share work earlier and share data. An important finding, which led the European Commission to support the principles of data that is findable, accessible, interoperable and reusable (FAIR), was the financial articulation of the opportunity cost of not having FAIR data: an estimated EUR10.2 billion for the European science system and EUR16 billion for the wider science system (Burgelman et al., 2019, p. 4). Policies that support open research have a clear financial benefit.

In thinking of who might lose power in a greater shift toward OS, journals that have enjoyed a position of exclusivity come to mind. A bold article published in the South African Journal of Industrial Psychology provides journal editors with several policy improvements that would enhance the journal's credibility and transparency (Efendic and Van Zyl, 2019). The authors proposed that journals that take the lead in OS policies have an opportunity to develop standards for a new scientific practice, which would, presumably, position those journals as continued preferred publishing avenues. Indeed, journals with OS policies are likely to be viewed more favorably by those in practice, where the openness assures readers outside of academia of the integrity of the science. Such research is more likely to be used for the benefit of society, a strong motivator for the importance of OS practices (Aguinis et al., 2020).

#### Implementation

##### Power, value, ethics

The expression of power and values that policies represent is a theme built upon by Lilja (2020) in her article looking at policy implementation. She raises issues from the perspective of the principal-agent theory. She puts forward that as policies shift power bases and as they express values that the implementing parties might not share, there is a risk of powerlessness and meaninglessness, which would create policy alienation. It is crucial, claimed Ali-Khan et al. (2017), that policies are born from the bottom-up, at least in part. Without the buy-in of those who will be implementing or be affected by the policies, there is a risk of policy alienation, resulting in an unsuccessful policy.

Policymakers should consider readiness for the OS policy, including “awareness, practices, and the perceived benefits” (Ahmed et al., 2019, p. 2). An earlier study from Canada, for example, showed that key partners must shape any successful policy. Still, before that can be done, there should be agreement on expectations, boundaries and engagement mechanisms (Ali-Khan et al., 2018). Armeni et al. (2021) showed the vital role that OS communities have in engaging role players to this end. Policymakers would be wise to partner with these communities to work toward their common goal.

However, not all stakeholders in the field of OS have a common goal. There is an evident tension between OS and the need to exploit findings for commercial gain. Industry partners have long relied upon researchers to provide sound conclusions on which to build products that meet consumer needs, but if those self-same researchers are pushed to share the findings openly, then the industry partners are left in a challenging position^1^.

There is more tension than clarity in this area, according to Chataway et al. (2017), more questions than answers. Policymakers would be wise to pay heed to this as it has possible implications within research as well–for example, in the cases given by Levin and Leonelli (2017). In one case, they discuss whether a particular piece of software, which is both a tool and a product of research, should be made openly available. Staunch supporters of the open movement would not hesitate to agree that it should. However, the researchers are using the software as bait to collect other datasets, and make those datasets available to other researchers, which is a far greater boon to research. In such a case, a blanket policy for openness would not serve well. The second example makes this point more evident. Levin and Leonelli (2017) used the case of a researcher who used transgenic mice in their work. As was the case with the software, these mice are both a tool and a product of the research. Naturally, such mice cannot be made freely available. To do so would simply be unethical. The ethical debates of OS are well summarized by Beauvais et al. (2021), who encouraged policy actors to engage with them to ensure that “Open Science can achieve its full potential” (p. 5), a potential they continue “can be envisioned, metaphorically, as a marathon, not a sprint.”

##### Implementation frameworks

**Figure 3:**
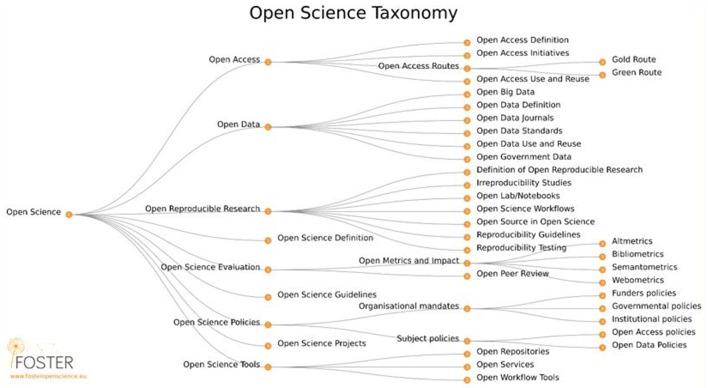
Open Science Taxonomy from Pontika et al. (2015). Figure available at https://doi.org/10.6084/m9.figshare.1508606.v3.

Looking beyond the navigation of the problematic issues that OS brings regarding power, values, and ethics, several valuable frameworks can be found in the literature to assist policymakers in framing robust instruments. Pontika et al. (2015) provided a thorough taxonomy of the OS landscape (see Figure 3). Not all OS policies will or should address all these areas; policymakers in Africa can use this taxonomy as a mapping tool to chart out pathways to ever more openness.

**Figure 4:**
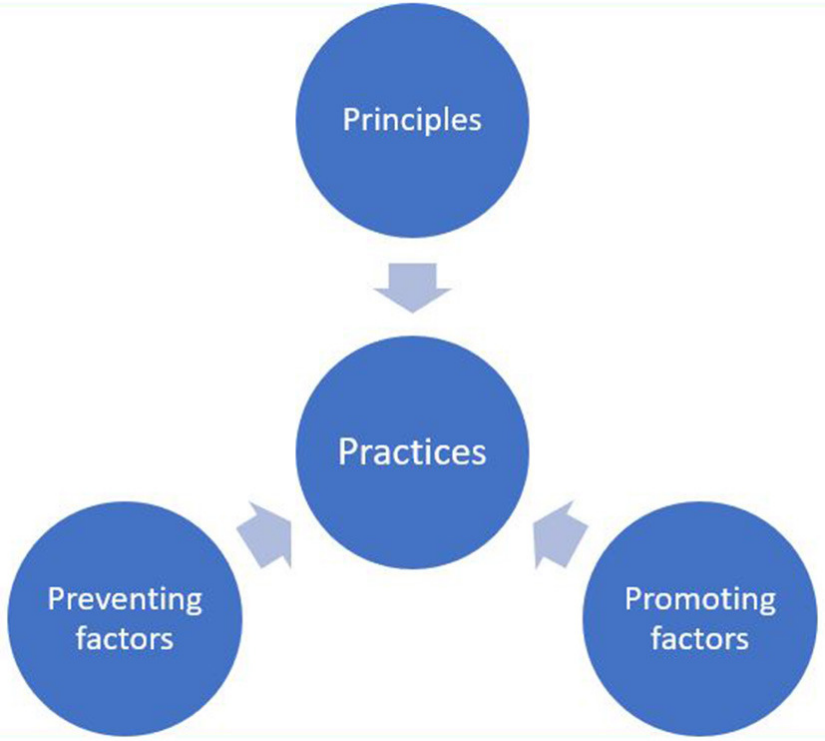
Conceptual model of governance of Open Science (adapted from Assist policymakers in framing robust instruments).

Morais et al. (2021) provided a helpful list of emergent areas of OS–including open collaborative tools, open physical labs, and crowdsource practices–which can be used to expand Pontika et al.'s taxonomy (2021). The list of emerging areas serves as a reminder to policymakers that the field of OS is by no means complete and that there will always be new areas and new issues to consider. Here, Vicente-Saez et al. (2020) provided a useful model for policymakers to bring new issues into their frame of thinking. Their model is adapted in Figure 4. It includes considerations around principles, promoting factors and preventing factors that all contribute to the practices within OS.

##### Examples

There is no shortage of examples of OS policies in the literature; those listed in Table 5 are a sample that emerged through the systematic review process. Policymakers who are disheartened in the challenging process can find solidarity in the cases outlined in these examples. For more examples of policies, without narratives, the Registry of Open Access Repository Mandates and Policies (ROARMAP) is a treasured resource.

**Table 5:** Examples of Open Science policies.

**Country/region**	**Detail**	**References**
Africa	University open data policies	Chiware, 2020
Albania	National science policies	Hasani et al., 2021
Botswana	National open policies	Ntlotlang, 2019
Canada	Research institute	Poupon et al., 2017
China	National open research policies	Li et al., 2022
China	National open research data policies	Zhang et al., 2021
Europe	Behind-the-scenes, regional policy	Burgelman, 2021
Finland	University library policies	Saarti et al., 2020
Hong Kong	National Open Science policies	Sharif et al., 2018
International	Funder policies	Borchert and Proudman, 2018
International	Funder policies	Clobridge and Hinsdale, 2018
International	Overview	Kuchma, 2017
International	Journal Open Science policies	Nosek et al., 2015
United States	National research data policies	Joseph, 2016

## Conclusion

The systematic review of literature highlighted what African policymakers should consider in terms of OS policy development in government and in academic and research institutions. It is clear from the review that what is important to policymakers in Africa is a consideration of the significance and value of OS and the accompanying policy frameworks. OS environments should be seen as more than technical problems and infrastructure development; they should also be seen as tools and mechanisms to solve broader societal problems. Levin and Leonelli (2017, p. 284) emphasized this point: “Openness is not only a technical problem to be solved but is also a social, cultural, and moral issue.”

Another critical point coming out of the analysis is the issues of OS policy readiness and, as Ahmed et al. (2019) pointed out, policymakers should consider the readiness for the OS policy, which would include awareness, practices, and the perceived benefits. Building onto this point are issues related to existing frameworks that should be considered in shaping the African OS policy environment. Existing frameworks, including tested taxonomies, are readily available and should be utilized. To strengthen these frameworks Beauvais et al. (2021, p. 5) advised that “technical considerations and responses to them must go hand in hand with ethical, legal and social ones.” In addition, when considering the uniqueness of the African continent, Vicente-Saez et al. (2020) provided a useful model that policymakers could use to bring new issues into their frame of thinking. This model is centered around practice that should consider aspects of principles, promotion and presentation factors.

Future policymakers can use the findings of this review to engage with policy stakeholders in a manner that will hopefully allow them to enact their values meaningfully. It can be used to examine policy failure and plot a path to the future.

## Author contributions

EC led the conceptualization, introduction, literature review, and conclusion. LS took the lead in methods, literature analysis, presentation of results, and discussion. All authors contributed to the article and approved the submitted version.

## Funding

Stellenbosch Business School has partially funded this work.

## Conflict of interest

The authors declare that the research was conducted in the absence of any commercial or financial relationships that could be construed as a potential conflict of interest.

## Publisher's note

All claims expressed in this article are solely those of the authors and do not necessarily represent those of their affiliated organizations, or those of the publisher, the editors and the reviewers. Any product that may be evaluated in this article, or claim that may be made by its manufacturer, is not guaranteed or endorsed by the publisher.

## Footnotes

^1^There have been movements that are not reported among the papers of this review and that reflect a growing openness in commercial spaces. These movements would stand in contrast to the findings here.
